# Assessment of Clinical Pharmacists' Assistance for Patients With Established Cardiovascular Diseases During the COVID-19 Pandemic: Insights From Southern India

**DOI:** 10.3389/fcvm.2020.599807

**Published:** 2020-12-03

**Authors:** Oliver Joel Gona, Ramesh Madhan, Sunil Kumar Shambu

**Affiliations:** ^1^Jagadguru Sri Shivarathreeshwara College of Pharmacy, Jagadguru Sri Shivarathreeshwara Academy of Higher Education and Research (JSS AHER), Mysore, India; ^2^Jagadguru Sri Shivarathreeshwara Medical College and Hospital, Jagadguru Sri Shivarathreeshwara Academy of Higher Education and Research (JSS AHER), Mysore, India

**Keywords:** COVID-19, cardiovascular diseases, clinical pharmacist, SARS-CoV- 2, corona virus 19

## Abstract

**Objectives:** We aimed to assess the clinical pharmacist-initiated telephone-based patient education and self-management support for patients with cardiovascular disease during the nationwide lockdown during COVID-19 pandemic.

**Methods:** A prospective single-center telephone-based cross-sectional study was conducted among patients at the Cardiology Department and its speciality clinic at a 1,800-bed tertiary care hospital in Southern India. A validated 8-item clinical pharmacist aided on-call questionnaire with two Domains was administered during and after lockdown (15 March and 8 June 2020). Clinical pharmacist-provided educational assistance on self-management practices were in accordance with the guidelines of Indian Council of Medical Research (ICMR) and World Health Organization. Comparisons was performed using sign test and association of responses were analyzed using the Goodman and Kruskal's gamma test. All the tests were two-tailed, *p* < 0.05 was considered to be statistically significant.

**Results:** Of the 1,080 patients, 907 consented with a response rate of (83.9%) and 574 (96.36%) patients were analyzed post-intervention. Majority of the patients were male (54.7%) and had Acute Coronary Syndrome [NSTEMI (42.10%), STEMI (33.92%) and Unstable Angina (9.86)]. The majority of subjects had at least two co-morbid conditions [(Type II Diabetes (48.33%), Hypertension (50.11%)] and were rural population (82.5%) as self-employed (43.1%) with a middle-class economy (31.6%). In the Domain-1 of checklist the awareness toward complications caused by COVID-19 in cardiovascular diseases (*Z* = −19.698, *p* = 0.000) and the importance of universal safety precautions enhanced after clinical pharmacist assistance [(*Z* = −8.603, *p* = 0.000) and (*Z* = −21.795, *p* = 0.000)]. In Domain-II of checklist there was a significant improvement in patients awareness toward fatal complications caused by COVID-19 (*Z* = −20.543, *p* = 0.000), maintenance of self-hygiene (*Z* = −19.287, *p* = 0.000), practice of universal safety precautions (*Z* = −16.912, *p* = 0.000) and self-isolation (*Z* = −19.545, *p* = 0.000). The results of our study population varied from baseline evaluation (41.7%, *n* = 907) to post-intervention (95%, *n* = 574) based on Literacy, employment status and economic status.

**Conclusions:** The proactive role of clinical pharmacists in providing instructional services in collaboration with cardiologist during the pandemic circumstances increased patients understanding and mitigated infection exposure among patients, health care professionals and also assuring the continuity of care in patients with established cardiovascular diseases.

## Introduction

In the last two decades, clustering and incidence of severe acute respiratory infections are one of the major threats to public health. Coronavirus disease (COVID-19) was first recorded in Wuhan, China, by the end of December 2019. Since then, COVID-19 has rapidly spread around the world. The COVID-19 was declared as a global pandemic on 11th March 2020 by the World Health Organization. COVID-19 has a major impact on public health and has a direct or indirect impact on social and economic activities. The exponential increase in the number of patients with COVID-19 in the past 6 months has overwhelmed health-care systems across the world. This is due to an inadequate understanding of the dynamic interplay of shifting epidemiology, publicity, pandemic prevention strategies, risk identification, and public health behavior (1). Cardiovascular disease is common comorbidity observed in patients infected with SARS or MERS (10 and 30% prevalence, respectively) (2). Currently, there is no promising evidence from randomized clinical trials (RCTs) that any potential therapy improves outcomes in patients with either suspected or confirmed COVID-19. Neither clinical trial data is supporting any prophylactic therapy.

The pre-existing cardiovascular disease seems to be linked with worse outcomes and increased risk of death in patients with COVID-19. Patients requiring intensive treatment had a significantly higher prevalence of chronic health conditions such as diabetes, cardiovascular and cerebrovascular disease (3). Moreover, COVID-19 itself can cause induce myocardial injury, arrhythmia, acute coronary syndrome and venous thromboembolism (4). Providing clinical care for patients with chronic cardiovascular disease and other comorbidities during pandemic times is challenging. Telehealth is an ideal platform to deliver clinical care during disasters and pandemics. Telemedicine negated the risk of COVID-19 exposure or transmission (5). In India, providing healthcare is a challenge, telemedicine ensures the safety of patients and health workers, especially when there is a risk of infection (6). India's digital health policy advocates the use of digital tools and focuses significantly on the use of telemedicine services, particularly at the grassroots level in the health and wellness Centers, where a mid-level provider/health worker can connect patients to doctors through technology platforms to provide timely and best possible care (7).

Citizens can make informed choices, defend themselves and comply with prescribed practices by focusing on what can be done during COVID-19 and when adequate resources are accessible, easily understood and communicated via reliable and accessible networks (8). Therefore, through collaboration between clinical pharmacist and cardiologist, we aimed to provide educational assistance regarding self-management practices in patients with existing cardiovascular diseases to mitigate exposure to COVID-19 infection.

## Methods

### Study Design and Participants

A prospective single-center telephone-based cross-sectional study was conducted among patients at the Cardiology Department and its speciality clinic at a 1,800-bed tertiary care hospital in Southern India serving 37 specialities. A validated 8-item clinical pharmacist aided on-call questionnaire with two Domains (Table 1) was administered during and after lockdown (15 March and 8 June 2020). Majority of the participants with acute coronary syndrome were the subset population of an ongoing clinical study and are currently being followed up. Clinical pharmacist-provided educational assistance on self-management practices was in accordance with the guidelines of Indian Council of Medical Research (ICMR) and World Health Organization.

**Table 1:** 8-item telemedicine questionnaire checklist of clinical pharmacists to assess awareness and knowledge regarding COVID-19 for patients with established cardiovascular diseases.

**S no**	**Questions**	**Response**	**Score**
**Domain-I: Assessment of awareness**			
1	Are you aware of the spread and impact of Novel corona virus 2019?	YES	1
		NO	2
2	Are you aware of the complications caused by Novel corona virus among patients with cardiovascular diseases?	YES	1
		NO	2
3	Are you aware of your present and past medical history	YES	1
		NO	2
4	Are you aware of the importance of universal safety precautions to prevent getting infected from Novel corona virus?	YES	1
		NO	2
**Domain-II: Assessment of knowledge**			
5	Do u know that Novel corona virus cause (SARS-nCoV-19) life threatening fatal complications among patients with cardiovascular complications and other co-morbid conditions?	YES	1
		NO	2
6	Do you know that self-isolation and maintenance of hygiene can aid in preventing infection from Novel corona virus cause (SARS-CoV-2)?	YES	1
		NO	2
7	Do you know how to follow universal safety precautions to prevent getting infected from Novel corona virus?	YES	1
		NO	2
8	Do you know that self-quarantine is a procedure followed by people who are at risk during epidemic?	YES	1
		NO	2

*The following questions in the domain-I and II are related to assess awareness and knowledge toward COVID-19 or SARS nCov-II infection in patients with established cardiovascular diseases by a clinical pharmacist through telephone*.

*This questionnaire is copyrighted and can be used as a tool for patients with established cardiovascular diseases without any changes and other clinical groups (can be modified accordingly) to assess awareness and knowledge about COVID-19 or SARS nCov-II infection*.

*The score for this questionnaire range between 8 and 16, participants are divided into high knowledge (8–12 score) and low knowledge (9–12) categories that has been derived by cumulative scores. Higher score (>12) for this questionnaire indicates that patients have lack of Awareness and Knowledge which indicates the need for educational assistance*.

### Reliability and Validity of the Questionnaire

Initially, the questionnaire was validated by selected faculty and research team using facial and content validation methods to ensure readability. To assess overall reliability, the internal consistency of individual items in each questionnaire domain was examined by the researchers. The questionnaire consists of two domains and eight questions pertaining to awareness and knowledge of subjects toward COVID-19. Each question consists of two responses which was scored as Yes is 1 and No is 2. The score for the questionnaire range between 8 and 16, for the purpose of identifying the status of awareness and knowledge, participants are divided into high knowledge (8–12) and low knowledge (13–16) categories that has been derived by cumulative score. Finally, the survey questionnaire was administered to patients by a clinical pharmacist to facilitate better understanding. Higher score (>12) for the questionnaire indicates that patients have lack of awareness and knowledge which indicates the need for educational assistance. This telemedicine questionnaire of clinical pharmacists to assess awareness and knowledge regarding COVID-19 for patients with established cardiovascular diseases was self-developed with scoring, there are no references identified to cite this conjecture.

### Sampling Method

This study followed a non-probability sampling method among the target population (subjects with established cardiovascular diseases at a tertiary care hospital).

### Outcome

The primary outcome of the study is to identify the impact of the clinical pharmacist-initiated educational guidance on COVID-19 pandemic among patients with established cardiovascular disease. The secondary outcome is to ensure continuity of care and compliance with the prescribed drugs.

### Statistical Analysis

Data were entered in MS Office Excel 2019 and analyzed using the IBM SPSS Statistics Version 25. Continuous variables were presented as mean ± standard deviation (SD). Categorical variables were presented as absolute numbers and percentages. Comparisons between baseline and post assistance scores among the individuals were performed using sign test, Association of Responses with socio-demographic variables were analyzed using the Goodman and Kruskal's gamma test. All tests were two-tailed, *p* < 0.05 was considered to be statistically significant.

## Results

Of the 1,080 patients contacted by telephone, the response rate at the baseline was 907 (83.9%) and 574 (63.28%) post-intervention. The majority (54.7%) of the study population were male and had at least two co-morbid conditions (44.56%) in the age group (61–80 years) (Table 2). The patients in the study had Acute Coronary Syndrome [NSTEMI (42.10%), STEMI (33.92%) and UA (9.86%)] followed by associated comorbidities as described in Table 3. The questionnaire developed was administered during and after nationwide lockdown. In the Domain-1 the patients were aware of the spread of COVID-19 (*p* = 0.000) and their current condition (*p* = 0.000). However, majority of them were not aware of the complications caused by COVID-19 among patients with cardiovascular diseases (*Z* = −19.698, *p* = 0.000) and the importance of universal safety precautions, their awareness enhanced after clinical pharmacist assistance [(Yes = 85.01 vs. 98.08%, No = 14.99 vs. 1.92%, Z = −8.603, *p* = 0.000) and (Yes = 11.84 vs. 94.94%, No = 88.15 vs. 5.05%, *Z* = −21.795, *p* = 0.000)]. In Domain-II regarding knowledge aspect majority of the patient's knowledge improved regarding fatal complications caused by COVID 19 (Yes = 22.12 vs. 95.98%, No = 77.87 vs. 4.01%, *Z* = −20.543, *p* = 0.000), the process of self-isolation, maintenance of self-hygiene (Yes = 33.97 vs. 99.12%, No = 66.02 vs. 0.88%, *Z* = −19.287, *p* = 0.000), the importance of universal safety precaution (Yes = 44.94 vs. 94.94%, No = 55.06 vs. 5.06%, *Z* = −16.912, *p* = 0.000) and regarding self-quarantine (Yes = 25.08 vs. 91.98%, No = 74.91 vs. 8.02%, *Z* = −19.545, *p* = 0.000) depicted in Table 4. The individual responses of the patients for every question at baseline was evaluated to correlate the association of sociodemographic variables with awareness and knowledge which demonstrated that the responses of the patients varied based on Literacy, employment status and economic status as represented in Table 5.

**Table 2:** Descriptive Summary of Demographics (*N* = 574).

**S. no**.	**Parameter**		**Summary^**#**^**
			**(*N* = 574)**	
1.	Age (in years)	21–40	30 (5.32%)
		41–60	254 (44.34%)
		61-80	257 (44.56%)
		81–100	33(5.76%)
2.	Gender	Male	314 (54.7%)
		Female	260 (45.23%)
3.	Literacy	Below high school	206 (36%)
		High school & above	287 (50%)
		Graduate & above	81 (14%)
4.	Economic status	Lower class	62 (10.7%)
		Upper-low class	254 (44.1%)
		Middle class	181 (31.6%)
		Upper class	77 (13.6%)
5.	Employment status	Salaried	111 (19.4%)
		Self-employed	248 (43.1%)
		Homemaker	215 (37.5%)
6.	Marital status	Married	530 (92.2%)
		Divorced/Widowed	51 (8.8%)
7.	Location	Urban	100 (17.5%)
		Rural	473 (82.5%)
8.	Smoking habit	Smokers	123 (21.5%)
		Non-smokers	450 (78.5%)
9.	Alcoholism	Occasional	153 (26.7%)
		Chronic	78 (13.5%)
		Non-alcoholics	401 (69.8%)
10	Time spent on call per patient	22.54 ± 11.23 min^a^	

# *Data represented as number (proportion), ^a^data represented as Mean ± SD, SD: Standard Deviation*.

**Table 3:** Clinical parameters.

**S. no**.	**Parameter**	**Summary^**#**^**	
		**(*N* = 574)**
1.	Acute coronary syndrome (ACS)	UA	57 (9.86%)
		NSTEMI	242 (42.10%)
		STEMI	195 (33.92%)
2.	Venous thromboembolism (VTE)	DVT	48 (8.4%)
		PE	33 (5.8%)
3.	T2DM	277 (48.33%)	
4.	HTN	288 (50.11%)	
5.	Kidney disease	72 (12.56%)	
6.	T2DM + HTN	292 (50.86%)	
7.	COPD	211 (36.73%)	
8.	Depression	22 (3.8%)	
9.	Atrial fibrillation	17 (2.9%)	

*T2DM, Type 2 Diabetes Mellitus; HTN, Hypertension; COPD, Chronic obstructive pulmonary disease; UA, Unstable angina; NSTEMI, non-ST segment elevation myocardial infarction; STEMI, ST-Elevation Myocardial Infarction*.

# *Data represented as number (proportion)*.

**Table 4:** Comparison of on call checklist responses before and after the clinical pharmacist assistance/intervention.

**S No**	**Questions**	**Baseline responses**	**Post assistance/intervention responses**			***Z*-value**	***P*-value**
			**Yes**	**No**	**Total**		
**Domain-I:**							
Q1	Are you aware of the spread and impact of Novel corona virus 2019?	Yes	487	0	487	−8.603	0.000^*^
		No	76	11	87		
		Total	563	11	574		
Q2	Are you aware of the complications caused by Novel corona virus among patients with cardiovascular diseases?	Yes	109	0	109	−19.698	0.000^*^
		No	390	75	465		
		Total	499	75	574		
Q3	Are you aware of your present and past medical history?	Yes	563	0	563	–	1.000
		No	0	11	11		
		Total	563	11	574		
Q4	Are you aware of the importance of universal safety precautions to prevent getting infected from Novel corona virus?	Yes	68	0	68	−21.795	0.000^*^
		No	477	29	506		
		Total	545	29	574		
**Domain-II:**							
Q5	Are you aware of the importance of universal safety precautions to prevent getting infected from Novel corona virus?	Yes	127	0	127	−20.543	0.000^*^
		No	424	23	447		
		Total	551	23	574		
Q6	Do u know that Novel corona virus cause (SARS-nCoV-19) life threatening fatal complications among patients with cardiovascular complications and other co-morbid conditions?	Yes	195	0	195	−19.287	0.000^*^
		No	374	5	379		
		Total	569	5	574		
Q7	Do you know how to follow universal safety precautions to prevent getting infected from Novel corona virus?	Yes	257	0	257	−16.912	0.000^*^
		No	288	29	317		
		Total	545	29	574		
Q8	Do you know that self-quarantine is a procedure followed by people who are at risk during epidemic?	Yes	144	0	144	−19.545	0.000^*^
		No	384	46	430		
		Total	528	46	574		

* *Statistically significant p-value (2-tailed, < 0.05) has been obtained by performing Sign test*.

**Table 5:** Association of baseline responses with socio-demographic variables among study participants.

**Question**	**Response**	**Literacy**					**Employment status**					**Economic status**					
		**1**	**2**	**3**	**Gamma**	***p*-value**	**1**	**2**	**3**	**Gamma**	***p*-value**	**1**	**2**	**3**	**4**	**Gamma**	***p*-value**
Q1	Yes	138	270	79	−0.776	0.000^*^	83	230	177	−0.043	0.693	55	223	153	56	0.252	0.008^*^
	No	68	17	2			28	18	38			7	31	28	21		
Q2	Yes	13	70	26	−0.532	0.000^*^	20	49	40	0.000	0.999	35	208	157	66	−0.329	0.000^*^
	No	193	217	55			91	199	175			27	46	24	11		
Q3	Yes	198	285	80	−0.556	0.055^*^	99	233	197	−0.042	0.761	46	246	175	70	−0.310	0.047^*^
	No	8	2	1			12	15	18			16	8	6	7		
Q4	Yes	4	32	32	−0.767	0.000^*^	26	20	22	0.266	0.021^*^	38	227	171	70	−0.465	0.000^*^
	No	202	255	49			85	228	193			24	27	10	7		
Q5	Yes	21	70	36	−0.520	0.000^*^	32	41	54	−0.008	0.925	44	38	26	19	0.336	0.000^*^
	No	185	217	45			79	207	161			18	216	155	58		
Q6	Yes	51	102	42	−0.323	0.000^*^	24	74	97	−0.337	0.000^*^	29	101	106	41	−0.196	0.003^*^
	No	155	185	39			87	174	118			33	153	75	36		
Q7	Yes	67	175	15	−0.076	0.275	47	113	97	−0.025	0.720	26	77	102	52	−0.379	0.000^*^
	No	137	113	67			64	135	118			36	177	79	25		
Q8	Yes	48	82	14	0.014	0.864	22	63	59	−0.114	0.157	19	53	54	7	0.094	0.222
	No	158	205	67			89	185	156			43	201	127	70		

* *Statistically significant p-value has been derived from application of Goodman and Kruskal's gamma test*.

*Literacy: 1: Below high school, 2: High school & above, 3: Graduate & above. Employment:1: Salaried, 2: Self-employed, 3: Homemaker. Economic status: 1: Lower class, 2: Upper-Low class, 3: Middle class, 4: Upper class*.

## Discussion

Pandemics and epidemics are a widespread problem then and now as COVID-19. During such periods, people in the community face several challenges. Lack of awareness and consciousness often leads to an uneasy attitude which could adversely affect the patients with established cardiovascular complications. Different stakeholders in their respective countries are working together to “flatten the curve” by joint prevention initiatives led by the WHO. With a practically sufficient global lockdown, Pharmacists appear to be the first contact point for meeting the health requirements of the public (8).

We studied the role of clinical pharmacists' assistance for patients with established cardiovascular diseases during the COVID-19. The principal findings in our study at initial assessment were (1) Most of the patients were aware of their medical condition (CVD and comorbidities), (2) Most of the patients were aware of SARS-CoV-2 (COVID-19) infection, (3) majority of the patients were unaware of fatal complications caused by COVID-19 and association of COVID severity with CVD and comorbidities, (4) most of them were unaware of the importance of universal safety precautions, (5) majority of them don't know that self-quarantine is a procedure followed by people who are at risk during the epidemic.

Pharmacists continue to play their role in promoting continuity of pharmaceutical care, as well as supporting governments for disseminating information on precautions related to COVID-19 spread (13). Pharmacists are an integral part of health care performing exceptional roles in past pandemics and health crises, with some, such as Ebola and Zika, posing global health security risks (9). In this study after assessing the awareness and knowledge we provided educational assistance which helped our patients to gain (1) knowledge regarding fatal complications caused by COVID 19, (2) the process of self-isolation, (3) maintenance of self-hygiene, (4) the importance of universal safety precaution and (5) regarding self-quarantine practice. The Chinese Centre for Disease Control and Prevention recently published the largest COVID-19 case series in mainland China; the overall fatality rate was 2.3% (1,023 deaths among 44,672 confirmed cases), but the mortality rate in patients with underlying CVD reached 10.5% (10). However, these results emphasize the potential risk of fatality in our patients with established cardiovascular disease and provide evidence regarding the need for intensive treatment on the infection (11).

The other measure which has been mentioned a lot in recent weeks is hand hygiene. The World Health Organization (WHO) regards handwashing with soap and water and friction with hydroalcoholic gel as the most effective measures for the prevention of infections and antimicrobial resistance (12). Research in major public universities following the H1N1 influenza pandemic reported inadequate compliance with preventive measures, such as residence at home when the virus is ill to prevent transmission in 2009 linked to the results of this study (14). Researchers can work with public agencies/health departments to set up information and awareness centers through participatory groups that may have significant population effects. Our study helped to implement preventive measures, such as isolation, quarantine and community confinement, early identification of cases, social assistance and the provision of patient-specific instructions.

As a consequence of COVID-19, the need for social distancing forced us to use all the resources in our toolbox, and telehealth is one of them that accelerated its adoption globally (15). Telehealth strategies should be encouraged with a view to increasing access and providing care to the patients with chronic diseases to promote continuity of their care which made us adapt the new normal practices. We need to make a conscious effort to avoid any possible worsening of the digital divide, and the government needs to take that responsibility in this case (16).

What was wonderful about it was during this pandemic when our patients are generally considered to be at greater risk of having more severe COVID-19 disease and when they have been asked to stay at home, through virtual visits, we are still able to maintain their continuity of care, evaluate their COVID-19 awareness and knowledge, and provide instructional assistance and mitigate their exposure to infection. Overall, the results reflect what might develop into a new standard of future health care, particularly during contagious outbreaks (17). In this difficult time, hopelessness is the mother of acceptance, virtual practices have become a new normal. But hopefully, as we emerge from this pandemic, the telemedicine infrastructure will remain and benefit those in need.

### Strengths and Limitations

During the times of stretched clinical resources due to COVID-19, our research results helped to add new ways to reduce COVID-19 spread in patients with established cardiovascular diseases. Although our study is a single-center study involving a clinical pharmacist, despite its limitations, the results in our study suggest that the extended role of the clinical pharmacist may also be beneficial to other clinical groups. In addition, more awareness amongst the study patients could also be attributed to govt initiated awareness programmes on COVID-19 (18). The results of this study may not be generalizable beyond India due to differences in clinical pharmacist practice worldwide.

## Conclusions

The clinical pharmacist may, however, play a pro-active role in promoting patient-specific treatment decisions by serving as a resource for physicians and other health care professionals to mitigate adverse events caused by SARS nCoV-2 infection in patients with established cardiovascular disease. The enhanced role of clinical pharmacists in providing instructional services should mitigate infection transmission during the COVID-19 pandemic.

## Data Availability Statement

The original contributions presented in the study are included in the article/supplementary materials, further inquiries can be directed to the corresponding author/s.

## Ethics Statement

The institutional ethics committee has approved this study and participants have been informed of the purpose of the study before participating and voluntary consent is obtained virtually. All procedures performed in this study involving human participants were consistent with the Declaration of Helsinki 1964 and its subsequent amendments or comparable ethical standards.

## Author Contributions

All authors listed have made a substantial, direct and intellectual contribution to the work, and approved it for publication.

## Conflict of Interest

The authors declare that the research was conducted in the absence of any commercial or financial relationships that could be construed as a potential conflict of interest.
